# Performance Evaluation of a Cicada-Inspired Subsoiling Tool Using DEM Simulations

**DOI:** 10.3390/biomimetics9010025

**Published:** 2024-01-03

**Authors:** Xuezhen Wang, Ruizhi Du, Lingxin Geng, Hanmi Zhou, Jiangtao Ji

**Affiliations:** 1 College of Agricultural Equipment Engineering, Henan University of Science and Technology, Luoyang 471000, China; xzwang@nwsuaf.edu.cn (X.W.);; 2 Collaborative Innovation Center of Machinery Equipment Advanced Manufacturing of Henan Province, Luoyang 471000, China

**Keywords:** biomimetic method, draught force, energy consumption, soil disturbance, subsoiling

## Abstract

Subsoiling practice is an essential tillage practice in modern agriculture. Tillage forces and energy consumption during subsoiling are extremely high, which reduces the economic benefits of subsoiling technology. In this paper, a cicada-inspired biomimetic subsoiling tool (CIST) was designed to reduce the draught force during subsoiling. A soil–tool interaction model was developed using EDEM and validated using lab soil bin tests with sandy loam soil. The validated model was used to optimize the CIST and evaluate its performance by comparing it with a conventional chisel subsoiling tool (CCST) at various working depths (250–350 mm) and speeds (0.5–2.5 ms^−1^). Results showed that both simulated draught force and soil disturbance behaviors agreed well with those from lab soil bin tests, as indicated by relative errors of <6.1%. Compared with the CCST, the draught forces of the CIST can be reduced by 17.7% at various working depths and speeds; the design of the CIST obviously outperforms some previous biomimetic designs with largest draught force reduction of 7.29–12.8%. Soil surface flatness after subsoiling using the CIST was smoother at various depths than using the CCST. Soil loosening efficiencies of the CIST can be raised by 17.37% at various working speeds. Results from this study implied that the developed cicada-inspired subsoiling tool outperforms the conventional chisel subsoiling tool on aspects of soil disturbance behaviors, draught forces, and soil loosening efficiencies. This study can have implications for designing high-performance subsoiling tools with reduced draught forces and energy requirements, especially for the subsoiling tools working under sandy loam soil.

## 1. Introduction

Energy requirements for crop production gradually increase because of the increasing population, which brings various heavier pieces of machinery to farmland [1]. The application of a conventional tillage system and frequent operations using different heavy agricultural machinery on farms generally results in soil compaction, which is an important issue in agricultural mechanization for the sustainable production of food [2,3,4,5,6]. Subsoiling practice is generally used to loosen the compacted soil, improve the growing conditions of roots, and restore yields of crops [7,8,9,10,11,12]. The practice mainly includes biological, natural, mechanical, and chemical methods [3,4,13]. Both natural soil recovery and biological methods need to experience a long period of time, and crop yields could be negatively affected during this period. Crop growth is not significantly affected by the chemical method. Moreover, the environment may be polluted by additional application of fertilization. By contrast, the mechanical method is widely used and can loosen soil and eliminate soil compaction within a short-term process [4]. The method could restore soil structure and improve soil properties [3,14]. However, draught forces and consumed energies during the subsoiling process are generally three to five times those of harvesting tools and seeders [15], which seriously reduces the economic benefits of subsoiling technology.

Bionics is the science of applying the characteristics of various creatures (animals, plants, and microbes) to modern technical equipment and creating new technologies [3,16,17,18]. Recently, various biomimetic methods have been substantially employed in the design of different agricultural soil-engaging tools because of the good structures of some creatures with lower tillage forces [19,20,21,22]. Bai et al. (2016) designed a new subsoiling tool with a badger claw-inspired shank; results showed that the draught forces were reduced by 3.01–7.29% compared with the conventional subsoiling tool with an arc-shaped shank (CSTA) [23]. In the study by Guo (2019), a biomimetic shank was developed based on the claw curve of the oryctolagus cuniculus, and draught forces were 6.4–8.2% lower than the CSTA [19]. A study by Li (2016) found that the design of a bear claw-inspired subsoiling tool reduced draught forces of the CSTA by 7.8–12.8% due to the decrease in soil disturbance [20]. The draught forces of biomimetic subsoiling tools were more or less reduced compared with conventional subsoiling tools. However, only the organ shapes of various animals were used as bionic prototypes in the design of subsoiling tools in the existing studies [19,20,23]. The movement modes of animals’ organs, which are essential in loosening the soil for animals, have rarely been considered in accordance with our previous review [5]. This hinders further decrease in draught forces and consumed energies of biomimetic subsoiling tools [5].

In the past few years, the discrete element method (DEM) has been utilized to investigate soil–tool interactions, which allows results to be obtained efficiently, such as the simulation of a cutting blade [24,25,26], the simulation of a sweep [27,28,29], the simulation of a harvesting tool [30,31], and the simulation of a subsoiler [6,32,33]. The above studies have proven that DEM is an effective and emerging advanced technology to evaluate tool performance in a timely manner.

It seems impossible that designing a soil-engaging tool inspired by only organ shape will give the lowest draught force, as the movement behaviors of some creatures’ organs (e.g., head, skin) are very essential in moving through the soil with small tillage forces. However, the combined effects of the organs’ shape and motion have rarely been included in designing a biomimetic subsoiling tool in previous research [5]. Therefore, the objectives of this study were to (1) design a biomimetic subsoiling tool bioinspired by both the profile and motion of the cicada head, (2) develop a soil–subsoiling tool interaction model by EDEM (Experts in Discrete Element Modeling) software and validate it using both measured draught force and soil disturbance behaviors, and (3) optimize the cicada-inspired subsoiling tool (CIST) and evaluate its performance by comparing it with a conventional chisel subsoiling tool (CCST) at various working depths and speeds in sandy loam soil.

## 2. Methodology

### 2.1. Biomimetic Design of the Cutting Share of the Subsoiling Tool

**Figure 1:**
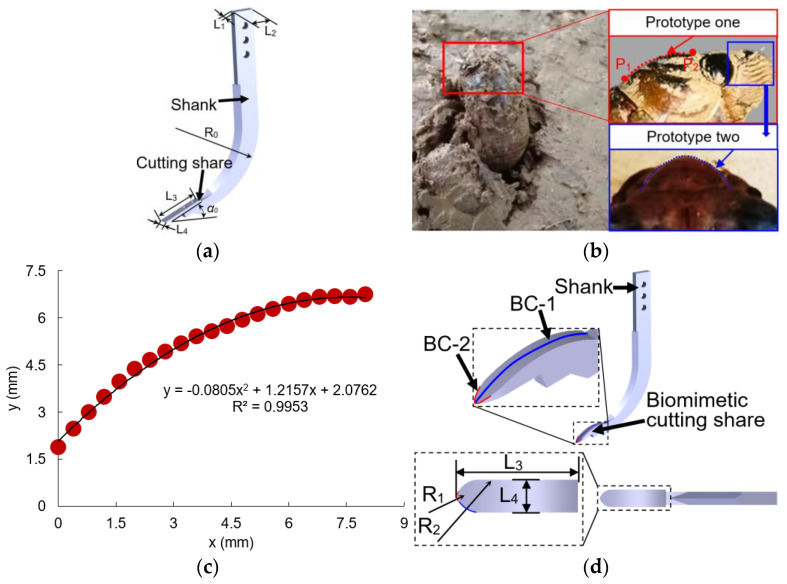
The (**a**) structure of a conventional chisel subsoiling tool, (**b**) prototypes of biomimetic cutting share, (**c**) fitting curve of prototype one, and (**d**) axonometric drawing and top view of a subsoiling tool with biomimetic cutting share (BC stands for biomimetic curve; L_1_ = 30 mm, shank width; L_2_ = 80 mm, shank thickness; L_3_ = 165 mm, share length; L_4_ = 40 mm, share width; α = 23°, share rake angle; R_0_ = 320 mm, shank curvature; R_1_ = 4 mm, radius of biomimetic share curve one; R_2_ = 40 mm, radius of biomimetic share curve two).

A conventional subsoiling tool generally includes a shank and a cutting share in accordance with the Chinese Standard (JB/T 9788-1999) (Figure 1a). The cutting share of a subsoiling tool generally moves forward linearly and is employed to break the soil and force the soil to fail. The draught force of the cutting share is the main source of the draught force of a subsoiling tool [14], and the structure of the cutting share has a significant impact on the tool’s draught force and soil disturbance [18,32,34]. The cicada is a soil animal that optimizes itself constantly over a long period of evolution. Thus, it gradually adapts to the soil environment and can freely move through the soil. The head shape of a cicada plays a key role in breaking soil during movement (Figure 1b). Moreover, the linear movement of the cicada head in the soil is very similar to the cutting share of a subsoiling tool during subsoiling operations. Thus, a cicada head was used as the prototype in the design of biomimetic cutting share.

**Figure 2:**
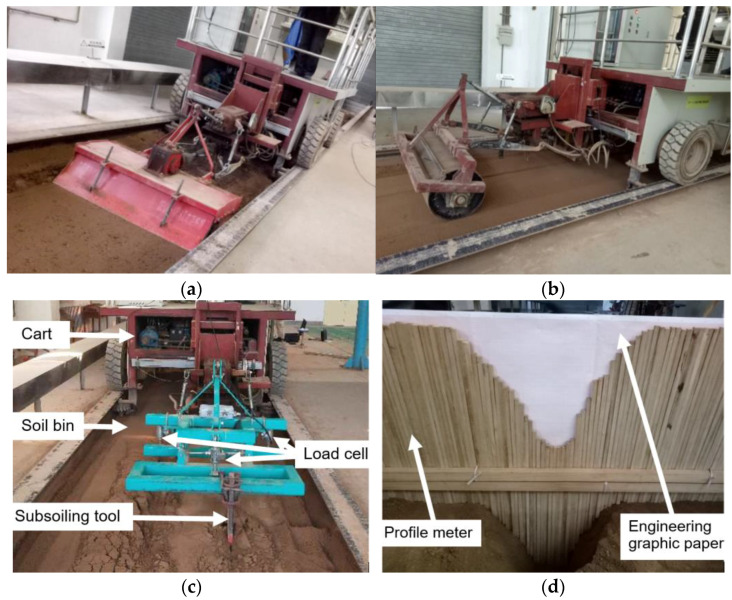
Lab soil bin test: (**a**) rotary tillage of the soil, (**b**) soil compaction, (**c**) subsoiling test, (**d**) soil disturbance profile measurement.

The lateral boundary curve of the cicada’s head was extracted using the following method: (1) marking two random points on the lateral profile of the cicada’s head (i.e., P_1_ and P_2_) and measuring the real distance between the two points using a vernier caliper (i.e., D_a_) (Figure 1b); (2) taking an image in the direction perpendicular to the plane where the lateral profile of the cicada’s head was located; (3) importing the image into the AutoCAD software and measuring the distance between the two marked points (D_b_) using the dimensioning tool of the software; and (4) scaling the image proportionally using the zoom function of AutoCAD2016 (zoom ratio = D_a_/D_b_) to obtain the image with real dimensions. The coordinates of the lateral boundary curve of the cicada’s head were then collected and fitted using a parabola with an R^2^ (coefficient of determination) of >0.995 (Figure 1c). Similar methods have been reported in earlier studies, which determined the lateral boundary curve of the soil cone by collecting the coordinates of soil particles on the profile of the soil cone [35,36]. The fitted curve was then scaled proportionally and extended laterally to produce a bionic curved plane of biomimetic cutting share (Figure 1d); moreover, the zoom ratio was determined based on the length of standard cutting share (Chinese Standard JB/T 9788—1999) (Figure 1a). The tip curve of the cicada’s head can be described by two arcs. To reduce the wear rate of cutting share, the tip curve of cutting share was designed by three sections of arcs; i.e., one arc with a radius of R_1_ and two arcs with a radius of R_2_. The cicada-inspired subsoiling tool with a conventional arc-shaped shank and a biomimetic cutting share is shown in Figure 1d.

### 2.2. Lab Soil Bin Tests

The soil tested in the lab soil bin was sandy loam soil. The soil moisture content, bulk density, yield strength, and void ratio were 19% (d. b.), 1350 kg m^−3^, 1.15 MPa, and 49.06%, respectively. The soil was prepared in three steps, including spraying water, rotary tillage, and compaction (Figure 2a,b) [14,32]. After soil preparation, a conventional chisel subsoiling tool (CCST) with an arc-shaped shank was selected based on the Chinese Standard (JB/T 9788—1999). The geometrical parameters of the tool are shown in Figure 1a. It was run in the soil bin at a constant speed of 1.5 m s^−1^ and a working depth of 300 mm in three replicates (Figure 2c). Draught forces during subsoiling were recorded by three load cells installed between the soil bin cart and the three-point hitch of the toolbar (accuracy: ±0.01 N) (Figure 2c). The soil disturbance area was measured using a 1500 mm wide profile meter that consists of 150 free-dropping wooden pins (Figure 2d). Initially, manual excavation of the disturbed soil was carefully performed after subsoiling; the profile meter was then placed at the top of the furrow and the pins in the profile meter automatically adjusted their vertical locations in accordance with the contour of the furrow; finally, furrow profiles were traced on the engineering graphic paper with a grid spacing of 1 mm, and the soil disturbance area was determined by the grid number in the furrow profile and the area of each grid (i.e., 1 mm^2^). The mean of the soil disturbance areas at three random locations was reported. Similar methods have been used in studies by Hang et al. and Wang et al. [14,32]. To ensure the stability of experimental data, the distance in the middle of the travel with a constant working speed was used for the measurement. The measured draught forces and soil disturbance behaviors in the lab soil bin were used to validate the following DEM model.

### 2.3. DEM Simulations

The DEM is a numerical method for examining tool–granular media interactions [27,35,37]. In this study, the subsoiling tool performance was evaluated using the DEM model developed with EDEM2021 software. The contact model between soil particles was a Hertz–Mindlin with a JKR model, which has been successfully used in many previous DEM studies [38,39,40]. Soil particles with spherical radii of 10 mm (nominal radii) were determined by comprehensively considering both the accuracy and solution time of the DEM simulations. Previous research implied that the generation of soil particles with fixed radii was not a realistic method for both particle packing and movement; however, this could be overcome by randomly generating soil particles in a radius range of 0.95–1.05 times the nominal particle radius [41]. Therefore, soil particles in the size range of 9.5–10.5 radii were generated in the DEM simulations. The DEM parameters can be grouped into material and interaction properties, which were determined by a combination of physical measurements, calibration, and data from other studies. The material parameters mainly consisted of the density, Poisson’s ratio, and the shear modulus of soil and the subsoiling tool (i.e., 65 Mn steel). Soil density was determined by actual measurement. The density and shear modulus of steel and the shear modulus and Poisson’s ratio of soil used in this study were published [33,38,42]. The interaction parameters mainly consisted of the surface energy between soil particles, the coefficient of restitution, and the coefficient of friction between materials. The coefficients of friction and rolling friction between soil and steel were measured by performing inclined plane tests where a tray filled with compacted and leveled soil was tilted until a piece of steel plane (or a spherical steel ball) commenced to move over the soil. The disturbance behaviors of sandy loam soil (e.g., angle of repose) are not significantly affected by the coefficients of restitution between materials [35], and they were determined by the average published data by Wang et al. and Zheng et al. [14,33]. The surface energy between soil particles was obtained from the published data in the study by Hu [38] with the same soil category and moisture content. The coefficients of friction and rolling friction between soil particles were calibrated by varying them until the simulated angle of repose (AOR) reached a close match of the measured value. The determined parameters are shown in Table 1.

**Figure 3:**
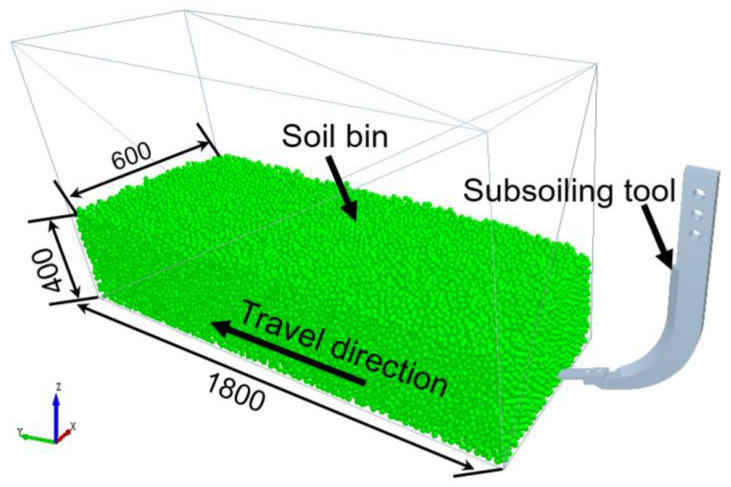
Subsoiling tool–soil interaction DEM model.

The virtual soil bin was 1.8 m long to ensure that the subsoiling tool could achieve its steady state on aspects of draught forces; the width and depth of the virtual soil bin were 0.6 m and 0.4 m, respectively, to avoid the effects of the bin walls’ edge on the movement of soil particles during subsoiling operations. The desired soil bulk density (i.e., bulk density of tested soil) in the virtual soil bin was achieved by pushing down the soil surface to a required depth. The 3D models of subsoiling tools were constructed using CATIA V5R20 software. After creating the virtual soil bin, the constructed tool model was imported into the EDEM, as shown in Figure 3.

### 2.4. Monitoring of Draught Forces and Soil Disturbance in the DEM

**Figure 4:**
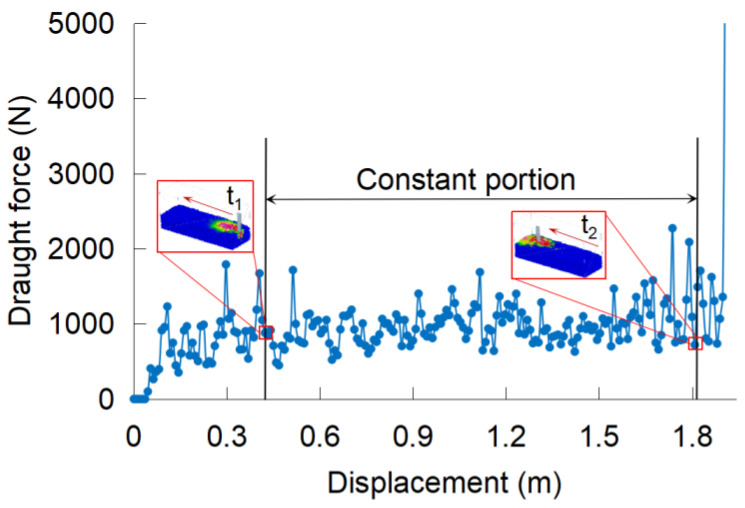
Draught forces vs. displacement during subsoiling.

After the validation, the DEM model was then used to optimize the biomimetic subsoiling tool and evaluate the performance of the optimized tool. Draught force is one of the most important indicators for subsoiling tools as it directly determines the tractor power requirement and energy consumption during tillage. The average draught force of every simulation run was taken over the constant portion of the force curve, which corresponded to the midsection (from moment t_1_ to moment t_2_) of the soil bin (Figure 4). Moment t_1_ stands for the moment when the subsoiler commenced and completely entered the soil bin and moment t_2_ stands for the moment when the cutting share commenced and left the soil bin.

**Figure 5:**
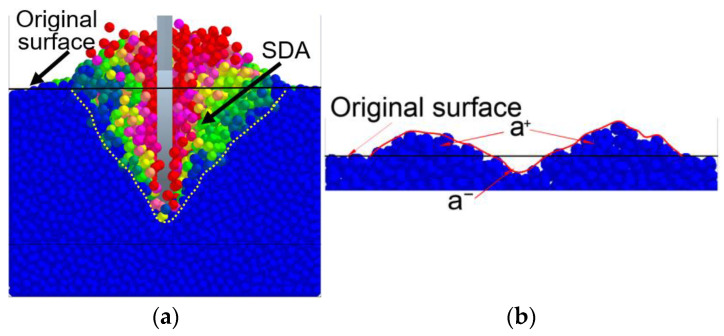
Soil disturbance: (**a**) soil disturbance area (SDA) and (**b**) positive (a+) and negative (a^−^) surface cross-sectional areas (SCAs) after subsoiling.

Soil disturbance area significantly affects the subsoiling effects on soil water infiltration and crop growth [13,43]. In the DEM model, soil particles with higher velocities around the tool indicated that they were disturbed during subsoiling. The profiles of soil disturbance around the tool during subsoiling were, therefore, obtained in accordance with the velocity field of soil particles around the tool (blue particles represented undisturbed soil) (Figure 5a). Better soil surface flatness is always desired for a subsoiling tool as it favors subsequent seeding operations. Surface cross-sectional areas after subsoiling could be used to evaluate the surface flatness, and it was defined as the sum of positive and negative surface cross-sectional areas (Figure 5b).

To optimize the cicada-inspired subsoiling tool (CIST), the above indicators of the CIST at varying rake angles (from 18° to 38°) were collected and analyzed. Moreover, to evaluate the tool performance of the new biomimetic design, draught force, surface cross-sectional area (SCA), soil disturbance area, and soil loosening efficiency from the CIST were compared with the conventional chisel subsoiling tool (CCST) at various working depths and working speeds. According to the Chinese Standard (JB/T 10295—2014), the working depth should not be less than 250 mm. The local working depth in subsoiling practices generally is not larger than 350 mm [44,45,46]. Moreover, the local working speed in subsoiling operations generally ranges from 0.5 m s^−1^ to 2.5 m s^−1^ [7,44,46]. The working depths of 250–350 mm and speeds of 0.5–2.5 m s^−1^ were, therefore, determined. Soil loosening efficiency (SLE) was calculated as follows [6].

(1) $$SLE=\frac{SDA}{F_{d}}$$

where SDA and *F_d_* stand for soil disturbance area (mm^2^) and the draught force during subsoiling (N).

## 3. Results and Discussion

### 3.1. Model Validation

**Figure 6:**
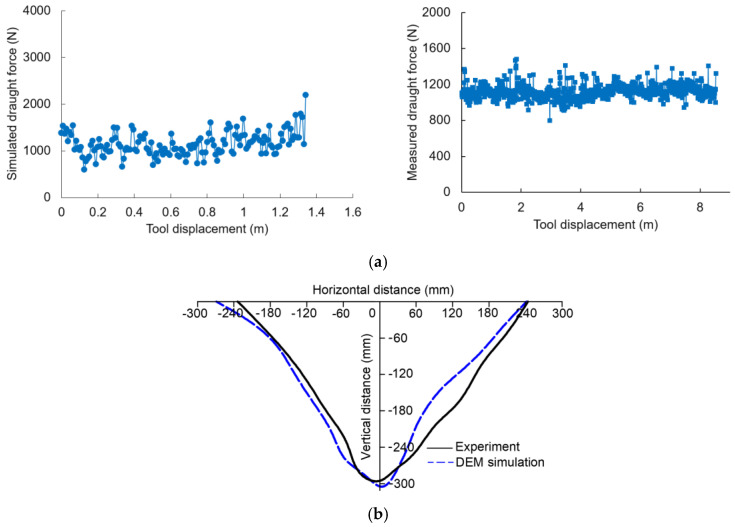
The (**a**) simulated and measured draught forces vs. displacement and (**b**) soil disturbance profiles from the DEM simulations and experiments.

The draught force of the conventional chisel subsoiling tool (CCST) over the tool displacement in the constant portion of the subsoiling travel is shown in Figure 6a. The force curve fluctuated over displacement, which is also the case in practice. The average draught forces from the DEM simulation and measurement were 1189 N and 1121 N, respectively, which gave a relative error of 6.07%. The low relative error indicated good agreement between the simulation and the measurement. In addition, soil disturbance profiles from the DEM simulation and experiment were basically consistent (Figure 6b); moreover, simulated and experimental soil disturbance areas were 71,210 mm^2^ and 73,306 mm^2^, which were also very comparable, as indicated by a relative error of <2.86%. The DEM model was again validated. The above results implied that the developed DEM model could be used to simulate soil–subsoiling tool interactions with good accuracy.

### 3.2. Biomimetic Subsoiling Tool Performance Affected by Rake Angle

**Figure 7:**
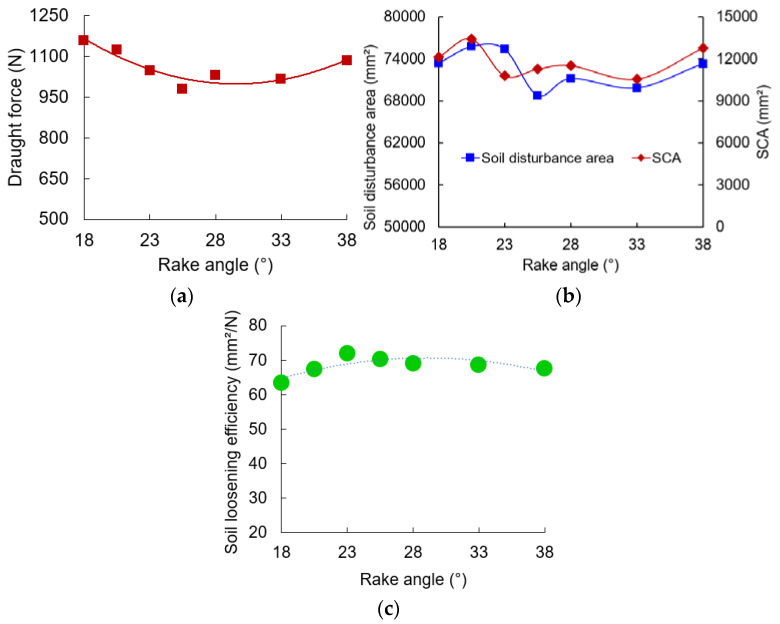
The performance of the cicada-inspired subsoiling tool (CIST) affected by rake angle: (**a**) draught force; (**b**) surface cross-sectional area (SCA) and soil disturbance area; (**c**) soil loosening efficiency.

For the DEM model with the JKR contact model, the simulated results from different repetitions did not vary, and the statistical analyses of these repeated results were, therefore, not performed. Rake angle is an essential factor affecting both the tool’s draught force and soil disturbance [45,47]. As shown in Figure 7a, draught forces of the cicada-inspired subsoiling tool (CIST) initially decreased and then increased with increasing rake angles; similar phenomena were also found in previous works [26,29,48]. Moreover, the apparently lower draught force was associated with a rake angle of 25.5°.

Both surface cross-sectional area (SCA) and soil disturbance area fluctuated with increasing rake angles from 18° to 38° (Figure 7b). The nonlinear effects of the rake angle on soil disturbance have been reported in the literature [26,45]. Comparably smaller SCA values were found at rake angles of 23–33°. Larger soil disturbance areas generally gave higher draught forces, as the rake angle ranged from 20.5° to 38°. For the CIST with a rake angle of 18°, draught force was relatively higher, while the soil disturbance area was smaller compared with the CIST with rake angles of 20.5–23°. Much smaller rake angles would result in the formation of a soil cone and soil back edge, which could reduce the cutting effects of the tool on soil and raise tillage forces in accordance with the studies by Hang et al. and McKyes [47,49]. The above research may explain the different variation trends between draught force and soil disturbance area at rake angles of 18–23°. With an increase in rake angle, soil loosening efficiency initially increased and then decreased (Figure 7c), and the CIST had comparably higher soil loosening efficiency at rake angles of 23–28°. The CIST with a rake angle of 25.5° would be the best choice for biomimetic design in terms of apparently lower draught force, comparably smaller SCA, and higher soil loosening efficiency.

### 3.3. Comparisons between Biomimetic and Conventional Subsoiling Tools

#### 3.3.1. Soil Disturbance Profile and Soil Disturbance Area

**Figure 8:**
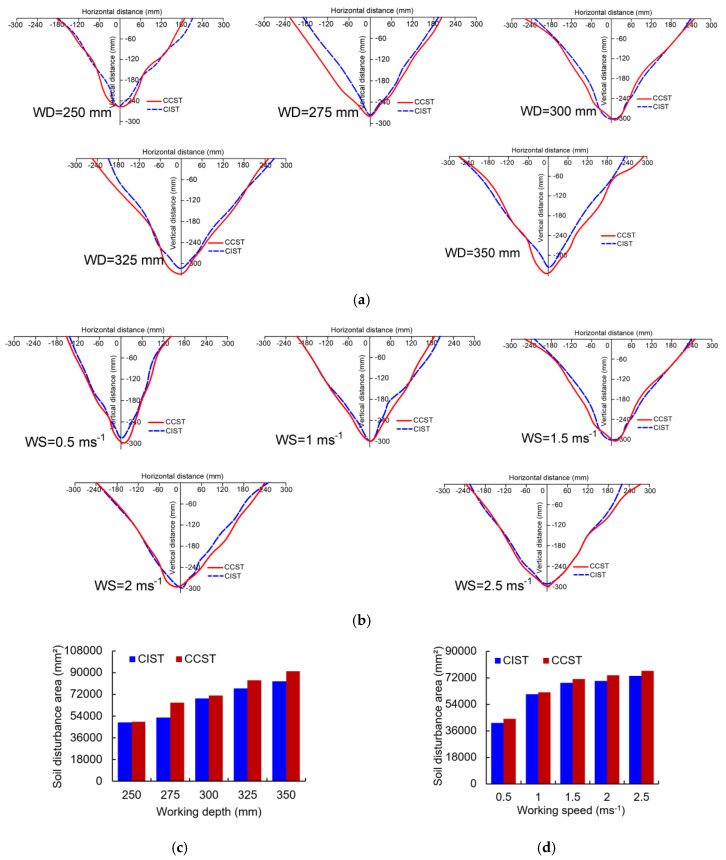
The soil disturbance profile at various working depths (WDs) (**a**) and working speeds (WSs) (**b**) and soil disturbance area affected by working depth (**c**) and working speeds (**d**) (CIST stands for cicada-inspired biomimetic subsoiling tool; CCST stands for conventional chisel subsoiling tool).

To evaluate the soil disturbance range, soil disturbance profiles of the CIST and CCST at various working depths and speeds were obtained, as shown in Figs. 8a and 8b. To further quantify soil disturbance amount, soil disturbance areas at various conditions were calculated (Figure 8c,d). An increasing working depth from 250 mm to 350 mm or working speed from 0.5 m s^−1^ to 2.5 m s^−1^ gave a larger soil disturbance area for both the CIST and CCST; moreover, working depth was more influential on soil disturbance area than working speed, especially for the CCST. Compared with the CCST, the soil disturbance areas of the CIST were 0.98–18.96% smaller at various working depths and 2.03–6.37% smaller at various working speeds. Less soil disturbance is generally favorable for reducing the draught force of soil-cutting tools [18,26].

#### 3.3.2. Draught Force

**Figure 9:**
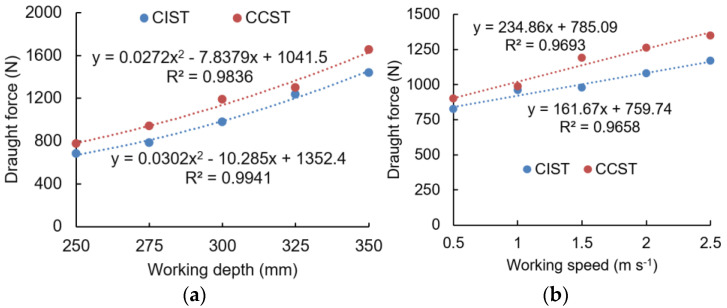
Draught force is affected by working depth (**a**) and working speed (**b**) (CIST and CCST stand for biomimetic and conventional subsoiling tools, respectively).

As shown in Figure 9a, both draught forces of the CIST and CCST were found to increase quadratically with working depths and coefficients of determination larger than 0.98. Moreover, the CIST had 4.93–17.70% lower draught force at various working depths compared with the CCST. By contrast, the effect of working speed on draught forces of the CIST and CCST had a linear increasing trend (Figure 9b); this was basically in line with the results by Yang et al., who reported that draught forces of a soil cutting blade were enlarged linearly when the working speed increased from 1 to 5 m s^−1^ [26]. The draught forces of the CIST were 2.82–17.70% lower than the CCST, as the working speed increased from 0.5 m s^−1^ to 2.5 m s^−1^. It was observed that working depth was much more influential on draught force than working speed, which agreed well with the study by Godwin [50].

The above results indicated that the cicada-inspired subsoiling tool (CIST) can reduce the draught force by 17.7% at various working depths and speeds; this design obviously outperforms the biomimetic designs by Bai et al., Guo, and Li, which have the largest draught force reductions of 7.29%, 8.2%, and 12.8%, respectively [19,20,23]. The main reason for the above performance improvement could be due to the fact that the combined effects of head shape and motion of cicada were considered in the biomimetic design of the subsoiling tool in this study; by contrast, only the organs’ shape (e.g., the claw) was considered in the above references. Therefore, the design of the CIST has a good potential to further reduce the draught forces and energy requirements of subsoiling operations.

#### 3.3.3. Surface Cross-Sectional Area (SCA)

**Figure 10:**
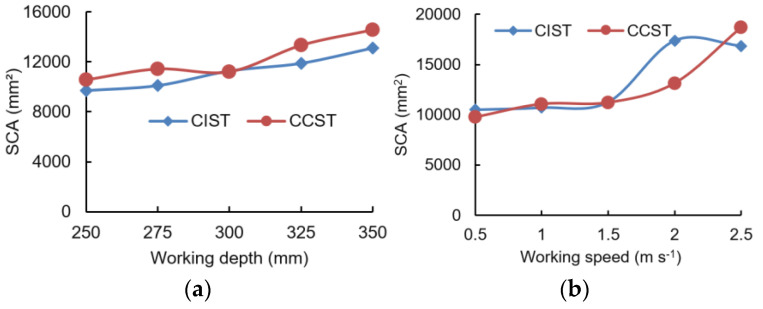
Surface cross-sectional area (SCA) affected by working depth (**a**) and working speed (**b**) (CIST and CCST stand for biomimetic and conventional subsoiling tools, respectively).

Overall, increasing the working depth or working speed gave a larger surface cross-sectional area (SCA) for both the CIST and CCST (Figure 10). SCAs for the CIST at various working depths were smaller than the CCST in most cases (Figure 10a). By contrast, SCAs for both the CIST and CCST were similar and quite small as the working speed increased from 0.5 m s^−1^ to 1.5 m s^−1^ (Figure 10b). With a further increase in working speed, the SCA increased rapidly. The above results indicated that soil surface flatness after subsoiling using the CIST was smoother at various depths than the CCST in most cases. Additionally, smaller working speeds (≤1.5 m s^−1^) are recommended to obtain a smoother soil surface flatness for both the CIST and CCST.

#### 3.3.4. Soil Loosening Efficiency

**Figure 11:**
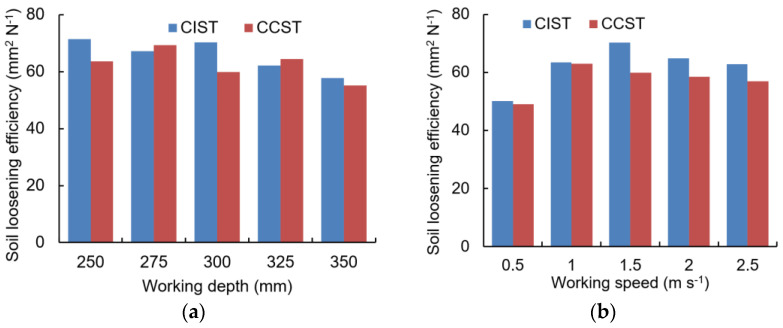
Soil loosening efficiency affected by working depth (**a**) and working speed (**b**).

The values of soil loosening efficiency (SLE) fluctuated for both the CIST and CCST with an increase in working depth from 250 mm to 350 mm (Figure 11a), which was supported by the study by Zeng et al. [6]. Moreover, increasing working depth gave a lower SLE. By contrast, SLE values of both the CIST and CCST initially increased and then decreased as the working speed increased from 0.5 m s^−1^ to 2.5 m s^−1^ (Figure 11b). Furthermore, soil loosening efficiencies of the CIST were 0.81–17.37% higher than the CCST at various working speeds.

## 4. Conclusions

In this study, a cicada head was used as the prototype of the biomimetic cutting share of a subsoiling tool to reduce the draught force requirement and energy consumption. A soil–subsoiling tool interaction DEM model was developed using EDEM and validated using the measured draught force, soil disturbance profile, and soil disturbance area of a conventional chisel subsoiling tool (CCST). The model was then used to optimize the cicada-inspired subsoiling tool (CIST) and evaluate its performance by comparing it with the CCST at various working depths (250–350 mm) and speeds (0.5 m s^−^^1^ to 2.5 m s^−^^1^). The following conclusions were drawn:

(1) The developed DEM model could be used to simulate soil–subsoiling tool interactions with good accuracy, as indicated by relative errors of <6.1% between simulated and measured draught forces and soil disturbance areas;
(2) Compared with the CCST, the draught forces of the CIST can be reduced by 17.7% at a working depth of 300 mm and working speed of 1.5 m s^−^^1^; this CIST design obviously outperforms the biomimetic designs by Bai et al. [23], Guo [19], and Li [20], which have largest draught force reductions of 7.29–12.8%. Therefore, the CIST has a good potential to further reduce the draught forces and energy requirements of subsoiling operations;
(3) Soil surface flatness after subsoiling using the CIST was smoother at various depths than the CCST. Soil loosening efficiencies of the CIST were 0.81–17.37% higher than the CCST at various working speeds.

Results from this study showed that the developed cicada-inspired subsoiling tool outperforms the conventional chisel subsoiling tool on aspects of both soil disturbance behaviors and draught forces. The tool’s performance was tested and validated only in a given soil condition, and future work will need to consider different soil conditions (e.g., soil categories).

## Figures and Tables

**Table 1:** Major parameters used in the DEM simulations.

Parameter	Unit	Value	Source
Poisson’s ratio of soil	Dimensionless	0.3	[42]
Shear modulus of soil	Pa	5 × 10^7^	[42]
Density of steel	kg m^−3^	7865	[33]
Poisson’s ratio of steel	Dimensionless	0.3	[33]
Shear modulus of steel	Pa	7.9 × 10^10^	[33]
Coefficient of static friction of soil–soil	Dimensionless	0.7	Calibrated
Coefficient of rolling friction of soil–soil	Dimensionless	0.225	Calibrated
Coefficient of restitution between materials	Dimensionless	0.6	[14,33]
Coefficient of static friction of steel–soil	Dimensionless	0.49	Measured
Coefficient of rolling friction of steel–soil	Dimensionless	0.06	Calibrated
Surface energy	J m^−3^	6	[38]
Rayleigh time step	s	2.2802 × 10^−4^	Calculated
Particle radii	mm	9.5–10.5	[41]

## Data Availability

The data reported in this study are contained within the article.
